# Supplementary material for: Randomized Clinical Trial of an Internet-Based Adolescent Depression Prevention Intervention in Primary Care: Internalizing Symptom Outcomes
Source: Int J Environ Res Public Health. 2020 Oct 22;17(21):7736. doi: 10.3390/ijerph17217736 (PMC7660174; doi:10.3390/ijerph17217736)
Supplement: Supplementary file 1 [file ijerph-17-07736-s001.pdf]

Table S1. Predictors[1] of missing 24-month CES-D_{10}, all participants.

	OR	(CI)		b	p
Intercept	--			-4.098	0.002
Boston (ref=Chicago)	0.546	(0.328	0.911)	-0.604	0.02
CATCH-IT (ref=HealthEd)	2.307	(1.457	3.651)	0.836	<0.001
Age at baseline, y	1.196	(1.026	1.393)	0.179	0.02
Male (ref=female)	1.528	(0.936	2.494)	0.424	0.09
Hispanic (ref=non-Hisp)	1.107	(0.609	2.010)	0.101	0.74
Non-white (ref=white)	0.834	(0.505	1.376)	-0.182	0.48
Mother's education (ref=college graduate)					
HS graduate/GED or less	3.274	(1.611	6.654)	1.186	0.001
Some college	0.932	(0.525	1.654)	-0.071	0.81
Parents' marital status (ref=married)					
Never married	0.994	(0.496	1.991)	-0.006	0.99
Divorced, separated, widowed	0.857	(0.480	1.531)	-0.154	0.60
Firstborn child (ref=not firstborn)	1.545	(0.971	2.458)	0.435	0.07
Past episode at baseline (ref=no past episode)	1.169	(0.705	1.939)	0.156	0.55
High CESD at screening or baseline (ref=no high CESD)	2.206	(1.035	4.700)	0.791	0.04

[a] From logistic regression model. Some observations were excluded from the model due to missing data for covariates: N=353.

Table S2. Predictors[1] of missing 24-month SCARED, all participants.

	OR	(CI)		b	p
Intercept	--			0.164	0.92
Boston (ref=Chicago)	0.142	(0.078	0.260)	-1.952	<0.001
CATCH-IT (ref=HealthEd)	1.852	(1.066	3.218)	0.616	0.03
Age at baseline, y	1.044	(0.865	1.259)	0.043	0.66
Male (ref=female)	0.971	(0.541	1.743)	-0.030	0.92
Hispanic (ref=non-Hisp)	1.410	(0.625	3.180)	0.344	0.41
Non-white (ref=white)	0.768	(0.411	1.433)	-0.265	0.41
Mother's education (ref=college graduate)					
HS graduate/GED or less	2.667	(1.059	6.719)	0.981	0.04
Some college	1.140	(0.564	2.305)	0.131	0.72
Parents' marital status (ref=married)					
Never married	0.634	(0.269	1.497)	-0.456	0.30
Divorced, separated, widowed	0.791	(0.388	1.612)	-0.234	0.52
Firstborn child (ref=not firstborn)	1.422	(0.802	2.519)	0.352	0.23
Past episode at baseline (ref=no past episode)	1.369	(0.721	2.601)	0.314	0.34
High CESD at screening or baseline (ref=no high CESD)	1.908	(0.829	4.387)	0.646	0.13

[1] From logistic regression model. Some observations were excluded from the model due to missing data for covariates: N=353.

Table S3. SCARED Moderator: Between-Group Comparisons: difference between CATCH-IT and HE simple slopes for time at selected levels of Parent CES-D[1.]

Parent CES-D10		Beta	SE	p-value
0.0	Difference	0.284	0.130	0.03
	CATCH-IT slope	0.037	0.092	0.69
	HE slope	-0.247	0.092	0.007
3.0	Difference	0.143	0.097	0.142
	CATCH-IT slope	-0.037	0.070	0.6017
	HE slope	-0.180	0.067	0.008
5.6	Difference	0.021	0.083	0.80
	CATCH-IT slope	-0.100	0.060	0.09
	HE slope	-0.122	0.057	0.03
9.0	Difference	-0.139	0.094	0.14
	CATCH-IT slope	-0.364	0.117	0.002
	HE slope	-0.225	0.083	0.007
27.0	Difference	-0.984	0.348	0.005
	CATCH-IT slope	-0.625	0.229	0.007
	HE slope	0.359	0.262	0.17

[1] Parent CES-D10 values shown are close to the minimum, the 25th, 50th, and 75th percentiles, and the maximum for this sample.

Table S4. SCARED Moderator: Between-Group Comparisons: difference between CATCH-IT and HE simple slopes for time at selected levels of PRPC[1.]

PRPC score		Beta	SE	p-value
1.0	Difference	1.248	0.482	0.01
	CATCH-IT slope	0.197	0.367	0.59
	HE slope	-1.052	0.312	<0.001
3.6	Difference	0.179	0.138	0.20
	CATCH-IT slope	-0.074	0.100	0.46
	HE slope	-0.252	0.095	0.008
4.0	Difference	0.014	0.124	0.91
	CATCH-IT slope	-0.116	0.086	0.18
	HE slope	-0.130	0.090	0.15
4.6	Difference	-0.233	0.157	0.14
	CATCH-IT slope	-0.178	0.109	0.10
	HE slope	0.055	0.114	0.63
5.0	Difference	-0.398	0.202	0.05
	CATCH-IT slope	-0.220	0.143	0.12
	HE slope	0.178	0.143	0.21

[1] PRPC values shown are close to the minimum, the 25th, 50th, and 75th percentiles, and the maximum for this sample.

Table S5. CES-D10 Moderator: Between-Group Comparisons: difference between CATCH-IT and HE simple slopes for time at selected levels of ADHD[1.]

ADHD score	Beta	SE	p-value
0.00 Difference	-1.198	0.432	0.006
CATCH-IT slope	-0.308	0.285	0.28
HE slope	0.891	0.325	0.006
0.50 Difference	-0.489	0.243	0.04
CATCH-IT slope	-0.247	0.160	0.12
HE slope	0.243	0.183	0.19
0.75 Difference	-0.135	0.198	0.50
CATCH-IT slope	-0.216	0.135	0.11
HE slope	-0.081	0.145	0.58
1.00 Difference	0.220	0.221	0.32
CATCH-IT slope	-0.185	0.155	0.23
HE slope	-0.405	0.157	0.01
2.00 Difference	1.637	0.616	0.008
CATCH-IT slope	-0.063	0.428	0.88
HE slope	-1.700	0.443	<0.001

[1] ADHD values shown are close to the minimum, the 25th, 50th, and 75th percentiles, and the maximum for this sample.

Table S6. CES-D10 Moderator: Between-Group Comparisons: difference between CATCH-IT and HE simple slopes for time at selected levels of ODCD[1.]

ODCD score	Beta	SE	p-value
0.00 Difference	-0.669	0.300	0.03
CATCH-IT slope	-0.294	0.190	0.12
HE slope	0.375	0.233	0.11
0.09 Difference	-0.455	0.246	0.06
CATCH-IT slope	-0.259	0.154	0.09
HE slope	0.196	0.191	0.31
0.17 Difference	-0.265	0.213	0.21
CATCH-IT slope	-0.229	0.138	0.10
HE slope	0.037	0.163	0.82
0.35 Difference	0.162	0.229	0.48
CATCH-IT slope	-1.454	0.454	0.002
HE slope	-1.615	0.490	0.001
1.25 Difference	2.297	0.958	0.02
CATCH-IT slope	0.184	0.708	0.80
HE slope	-2.113	0.646	0.001

[1] ODCD values shown are close to the minimum, the 25th, 50th, and 75th percentiles, and the maximum for this sample.

Table S7. CES-D10 Moderator: Between-Group Comparisons: difference between CATCH-IT and HE simple slopes for time at selected levels of PRPC[1].

PRPC score	Beta	SE	p-value
1.0 Difference	1.974	0.910	0.03
CATCH-IT slope	0.756	0.701	0.28
HE slope	-1.218	0.580	0.04
3.6 Difference	0.427	0.256	0.10
CATCH-IT slope	-0.149	0.184	0.42
HE slope	-0.576	0.178	0.001
4.0 Difference	0.188	0.232	0.42
CATCH-IT slope	-0.289	0.158	0.07
HE slope	-0.477	0.171	0.005
4.6 Difference	-0.169	0.300	0.57
CATCH-IT slope	-1.387	0.721	0.06
HE slope	-1.218	0.580	0.04
5.0 Difference	-0.407	0.386	0.29
CATCH-IT slope	-0.637	0.274	0.02
HE slope	-0.230	0.272	0.40

[1] PRPC values shown are close to the minimum, the 25th, 50th, and 75th percentiles, and the maximum for this sample.
